# High blocking temperatures for DyScS endohedral fullerene single-molecule magnets†

**DOI:** 10.1039/d0sc05265e

**Published:** 2020-11-02

**Authors:** Wenting Cai, Joshua D. Bocarsly, Ashley Gomez, Rony J. Letona Lee, Alejandro Metta-Magaña, Ram Seshadri, Luis Echegoyen

**Affiliations:** Department of Chemistry, University of Texas at El Paso 500 W University Avenue El Paso Texas 79968 USA echegoyen@utep.edu; Materials Research Lab, Materials Department, University of California Santa Barbara Santa Barbara California 93106 USA seshadri@mrl.ucsb.edu

## Abstract

Dy-based single-molecule magnets (SMMs) are of great interest due to their ability to exhibit very large thermal barriers to relaxation and therefore high blocking temperatures. One interesting line of investigation is Dy-encapsulating endohedral clusterfullerenes, in which a carbon cage protects magnetic Dy^3+^ ions against decoherence by environmental noise and allows for the stabilization of bonding and magnetic interactions that would be difficult to achieve in other molecular architectures. Recent studies of such materials have focused on clusters with two Dy atoms, since ferromagnetic exchange between Dy atoms is known to reduce the rate of magnetic relaxation *via* quantum tunneling. Here, two new dysprosium-containing mixed-metallic sulfide clusterfullerenes, DyScS@*C*_s_(6)–C_82_ and DyScS@*C*_3v_(8)–C_82_, have been successfully synthesized, isolated and characterized by mass spectrometry, Vis-NIR, cyclic voltammetry, single crystal X-ray diffractometry, and magnetic measurements. Crystallographic analyses show that the conformation of the encapsulated cluster inside the fullerene cages is notably different than in the Dy_2_X@*C*_s_(6)–C_82_ and Dy_2_X@*C*_3v_(8)–C_82_ (X = S, O) analogues. Remarkably, both isomers of DyScS@C_82_ show open magnetic hysteresis and slow magnetic relaxation, even at zero field. Their magnetic blocking temperatures are around 7.3 K, which are among the highest values reported for clusterfullerene SMMs. The SMM properties of DyScS@C_82_ far outperform those of the dilanthanide analogues Dy_2_S@C_82_, in contrast to the trend observed for carbide and nitride Dy clusterfullerenes.

## Abstract 2

Extremely high magnetic blocking temperatures (∼7.3 K) were observed for DyScS endohedral fullerene single-molecule magnets.

## Graphical abstract

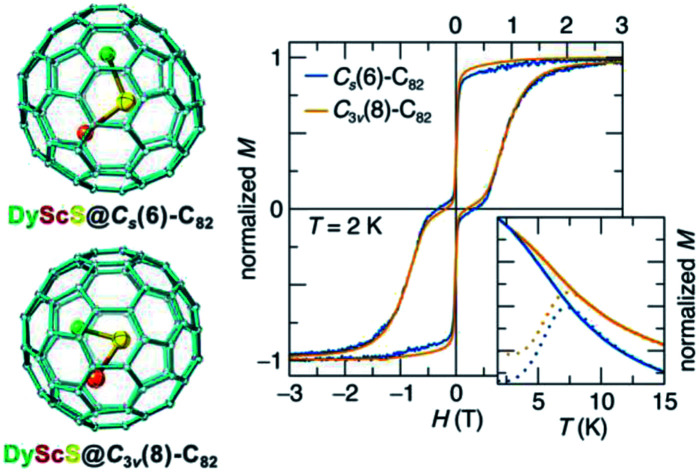

## Introduction

In magnetic molecules with bistable ground states and significant anisotropy, long-lived magnetic memory may be observed down to the single-molecule level. Such “single-molecule magnets” (SMMs) show great promise for applications such as ultrahigh density magnetic memory, spintronics, and quantum computing. Towards this goal, the major research objective in the field of SMMs is to design molecules with longer magnetic lifetimes at higher temperatures. One of the most promising strategies to achieve high-performing SMMs is by coupling magnetic lanthanide ions (usually Dy^3+^) to strong axial ligand fields. This approach has resulted in magnetic systems with energy barriers to relaxation nearing *U*_eff_ = 2000 K.^1–4^

One particularly interesting class of SMMs are the lanthanide endohedral metallofullerenes (EMFs).^1^ In these compounds, fullerenes encapsulate atoms or small clusters of atoms, often stabilizing unusual atomic arrangements that would not be possible in conventional molecules, and shielding those exotic states from environmental sources of decoherence. These unique conditions can lead to well-controlled, high-performing SMMs.^5^ Furthermore, EMFs present enticing opportunities for the assembly of precisely controlled nanoscale SMM devices *via* functionalization of the fullerene cage.^6^ The study of EMF SMMs began with the discovery of slow magnetic relaxation in DySc_2_N@C_80_, which shows a magnetic blocking temperature of up to *T*_B_ = 7 K.^7,8^ Subsequently, several other nitride, carbide, sulfide, and oxide clusterfullerenes (ECFs) have all shown slow magnetic relaxation.^5^ In these clusterfullerenes, the fullerenes stabilize short bonds between Dy and the nonmetallic element,^9^ resulting in strong axial fields generating thermal barriers to relaxation on the order of hundreds of kelvins, and even up to 1735 K.^8^

Given the high thermal barriers to relaxation observed in Dy-based EMFs, one may expect very high magnetic blocking temperatures and extremely long magnetic lifetimes at low temperatures. However, in most cases, the practical performance of a SMM is ultimately limited by through-barrier relaxation processes including quantum tunneling of magnetization (QTM), which causes the magnetic relaxation lifetimes to plateau at low temperatures. Finding ways to limit QTM is therefore of the upmost importance in the search for higher-performing SMMs. One promising approach is to introduce ferromagnetic exchange between two magnetic ions, as was demonstrated in the di-lanthanide clusterfullerene Dy_2_ScN@C_80_. Compared to DySc_2_N@C_80_, this compound was shown to exhibit suppressed QTM and a higher blocking temperature *T*_B_ = 8 K. For this reason, most of the recent work on EMF SMMs have been focusing on systems with two Dy atoms, such as Dy_2_S@*C*_s_(6)–C_82_,^10^ Dy_2_S@*C*_3v_(8)–C_82_,^10^ Dy_2_O@*C*_s_(6)–C_82_,^9^ Dy_2_O@*C*_3v_(8)–C_82_,^9^ Dy_2_O@*C*_2v_(9)–C_82_,^9^ Dy_2_C_2_@*C*_s_(6)–C_82_,^10^ Dy_2_TiC@*I*_h_(7)–C_80_,^11^ Dy_2_TiC_2_@*I*_h_(7)–C_80_,^11^ Dy_2_ScN@*D*_5h_(6)–C_80_.^12^ The most successful implementation of this strategy has been in pure dimetallofullerenes, such as Dy_2_@C_80_(CH_2_Ph), which hosts strong Dy–Dy ferromagnetic exchange along a radical Dy–Dy bond, leading to remarkable SMM behavior with *T*_B(100)_ = 18 K.^13^

However, suppression of QTM is also possible for single lanthanide compounds, if the magnetic ion is in a highly symmetric environment. In fact, in non-EMF SMMs, this approach has proven to result in the highest-performing SMMs.^3,14–18^ A computational study focusing on hypothetical monolanthanide oxide clusterfullerenes DyXO@C_82_ (X = Sc, Lu) has suggested that the Dy ligand field in these compounds, composed of oxygen and carbon from the fullerene, should provide a suitably symmetric environment to yield large thermal barriers to relaxation while simultaneously suppressing quantum tunneling.^19^ However, while Dy_2_O@C_82_ ^9^ and Dy_2_S@C_82_ ^10^ have been experimentally studied, their mono-lanthanide analogues have not.

In this study, we report the synthesis, isolation, structural characterization and SMM properties of new mixed metallic dysprosium-based sulfide clusterfullerenes, DyScS@*C*_s_(6)–C_82_ and DyScS@*C*_3v_(8)–C_82_. Both crystallographic analyses and electrochemical studies show that the replacement of one Dy by Sc exerts a noticeable influence on the conformation of the encapsulated cluster inside the fullerene cages. Remarkably, both isomers show open magnetic hysteresis loops at temperatures below 7 K, indicating SMM behavior. The magnetic blocking temperatures for both isomers are around *T*_B_ = 7.3 K, by far the highest blocking temperature for a sulfur-ligated Dy SMM,^10,20–22^ and among the highest blocking temperatures reported for a pristine EMF. Of particular note, this *T*_B_ far exceeds that of Dy_2_S@C_82_ (*T*_B_ = 2 K to 4 K).^10^ Analysis of magnetic relaxation times at zero field and under a moderate magnetic field suggests the presence of some degree of QTM at zero-field; nonetheless, the lifetimes are found to be much longer than those of Dy_2_S@C_82_, even at zero field. This result points to the promise of mono-dysprosium clusterfullerenes to achieve excellent SMM behavior.

## Results and discussion

### Preparation, purification and spectroscopic characterizations of DyScS@C_82_

**Fig. 1:**
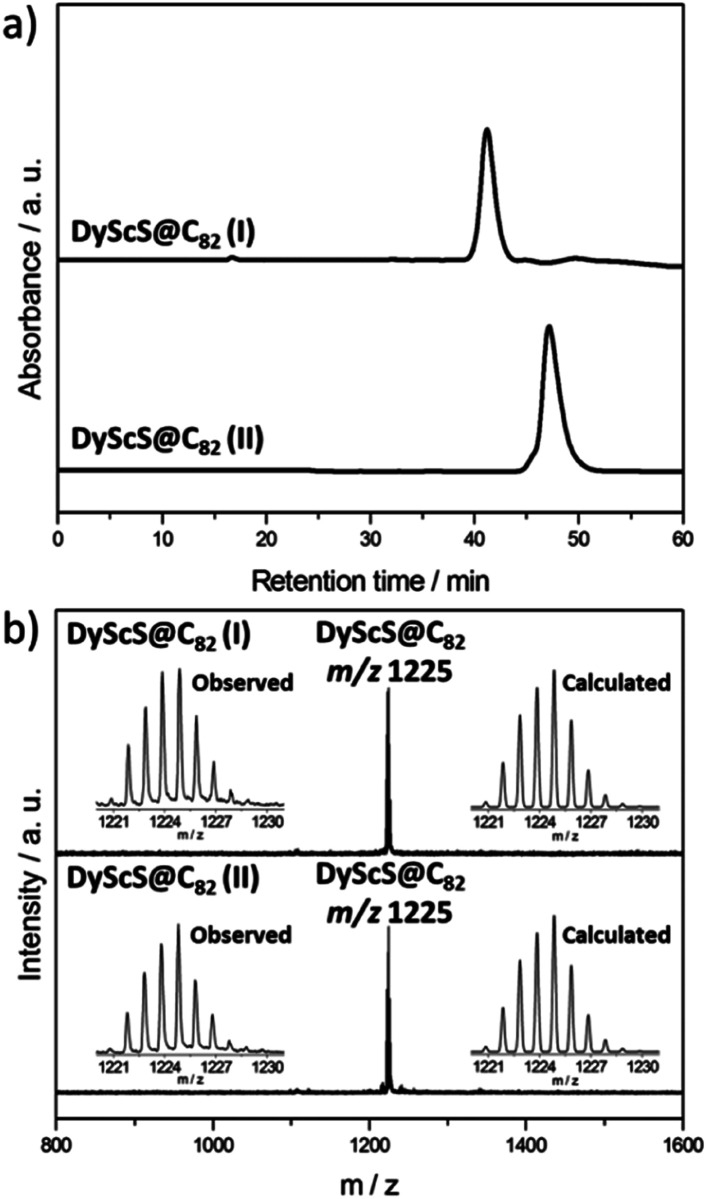
(a) HPLC chromatograms of purified DyScS@C_82_ (I, II) on a Buckyprep column with toluene as the eluent at a flow rate of 4 mL min^−1^; (b) the positive mode MALDI-TOF mass spectra and expansions of the experimental and theoretical isotopic distributions of DyScS@C_82_ (I, II).

Generally, the dimetallic sulfide clusterfullerenes were produced *via* two synthetic methods. Dunsch *et al.* introduced the sulfur source using solid guanidinium thiocyanate (CH_5_N_3_·HSCN), in addition to graphite powder.^23^ Using this method, only one isomer of Sc_2_S@*C*_3v_(6)–C_82_ was obtained as a minor product along with major products of Sc_3_N@C_80_ and Sc_3_N@C_78_. Our group introduced SO_2_ as the sulfur source, to produce sulfide cluster metallofullerenes as major products with a few minor products, such as oxide cluster metallofullerenes.^24–26^ In order to produce mixed-metallic sulfide clusterfullerenes, soots containing DyScS@C_2*n*_ clusterfullerenes were produced by a modified direct current arc-discharge method.^27^ As source material, Dy_2_O_3_, Sc_2_O_3_ and graphite powder were mixed in a weight ratio of 2.6 : 1 : 3, and packed in graphite rods. The arc synthesis was carried out under 210 torr He and 20 torr SO_2_ as the source of sulfur. The soot was then Soxhlet extracted with CS_2_ for 12 hours. As shown in Fig. S1,† DyScS@C_82_ and DyScS@C_84_ were obtained along with a family of Sc_2_S@C_2*n*_ endohedrals (2*n* = 82–90). The existence of Sc_3_N@C_80_ is attributed to the presence of a small leak in the reactor. Similar to the previously reported results with NH_3_, the formation of empty fullerenes was largely suppressed and a relatively high selectivity for the production of sulfide clusterfullerenes was observed with SO_2_. Multistage HPLC separation procedures were employed to isolate and purify them (see Fig. S2–S5, ESI†). The purity of the isolated DyScS@C_82_ (I, II) were confirmed by the observation of single peaks on the chromatograms with different columns and by the observation of single peaks in the MALDI-TOF mass spectra (Fig. 1 and S5†). Both compounds are reasonably pure isomers, as evident by the very different retention times on BuckyPrep column (Fig. 1).

To obtain additional structural information for DyScS@C_82_ (I, II), the Vis-NIR spectra were recorded (see Fig. S6, ESI†). The Vis-NIR absorption spectrum of DyScS@C_82_ (I) exhibits distinct absorptions at 868, 787, 717 and 635 nm, which are very similar to those reported for Sc_2_S@*C*_s_(6)–C_82_ ^24^ and Dy_2_S@*C*_s_(6)–C_82_.^10^ DyScS@C_82_ (II) exhibits only two absorptions at 883 and 662 nm, which resemble those reported for Sc_2_S@*C*_3v_(8)–C_82_ ^24^ and Dy_2_S@*C*_3v_(8)–C_82_.^10^ Based on the UV-Vis spectral observations, it is reasonable to assign the two C_82_ cages to DyScS@*C*_s_(6)–C_82_ and DyScS@*C*_3v_(8)–C_82_, respectively.

### Crystallographic characterization of DyScS@*C*_s_(6)–C_82_ and DyScS@*C*_3v_(8)–C_82_

**Fig. 2:**
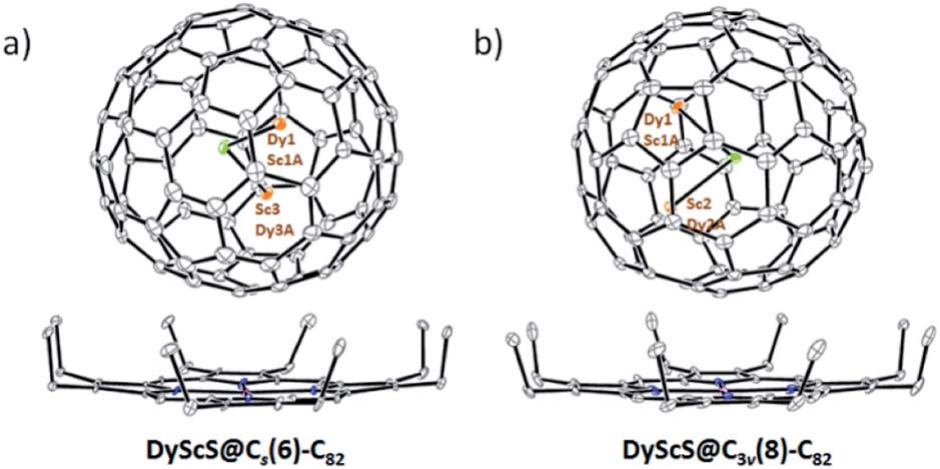
ORTEP drawing of (a) DyScS@*C*_s_(6)–C_82_·Ni^II^(OEP) and (b) DyScS@*C*_3v_(8)–C_82_·Ni^II^(OEP) with 10% thermal ellipsoids, respectively. Only the predominant DyScS clusters are shown, whereas minor sites and solvent molecules are omitted for clarity.

The molecular structures of DyScS@*C*_s_(6)–C_82_ and DyScS@*C*_3v_(8)–C_82_ were additionally established using single-crystal X-ray diffraction. Co-crystals for both compounds were obtained by layering a benzene solution of Ni^II^(OEP) (OEP = 2,3,7,8,12,13,17,18-octaethylporphyrin dianion) over a nearly saturated CS_2_ solution of the purified endohedral. Fig. 2 shows the structures of the fullerenes and their relative orientations with respect to the co-crystallized Ni^II^(OEP) molecules. The shortest fullerene cage to Ni^II^(OEP) contacts are 2.955 Å and 2.808 Å for DyScS@*C*_s_(6)–C_82_ and DyScS@*C*_3v_(8)–C_82_, respectively, which are typical distances for π–π stacking interactions between the fullerene and the porphyrin moiety.

Within the *C*_s_(6)–C_82_ and *C*_3v_(8)–C_82_ cage, both the Dy and Sc sites are disordered. It is difficult to distinguish Dy and Sc unambiguously for both cases because the electron densities at those positions are crystallographically similar. An alternate approach to assign the metals is based on the possible different Dy/Sc–S bond lengths in the DyScS cluster due to the ionic size of Sc (0.745–0.87 Å for Sc^3+^) and that of Dy (0.912–1.083 Å for Dy^3+^). Thus, the Sc–S bonds are expected to be slightly shorter in comparison with the Dy–S bonds, in good agreement with the reported Sc–S bonds (2.34–2.41 Å) for the Sc_2_S cluster and Dy–S bonds (2.43–2.51 Å) for the Dy_2_S cluster. However, the distribution of the metal–sulfide bond lengths in the DyScS clusters are not so different, making it difficult to exclude the overlap between Sc and Dy positions in DyScS@*C*_s_(6)–C_82_ and DyScS@*C*_3v_(8)–C_82_. Accordingly, we treat all metallic sites as overlapped Dy/Sc positions, similar to the procedure reported for DyEr_2_N@*I*_h_–C_80_ and DyEr@*C*_3v_(8)–C_82_.^28,29^

**Fig. 3:**
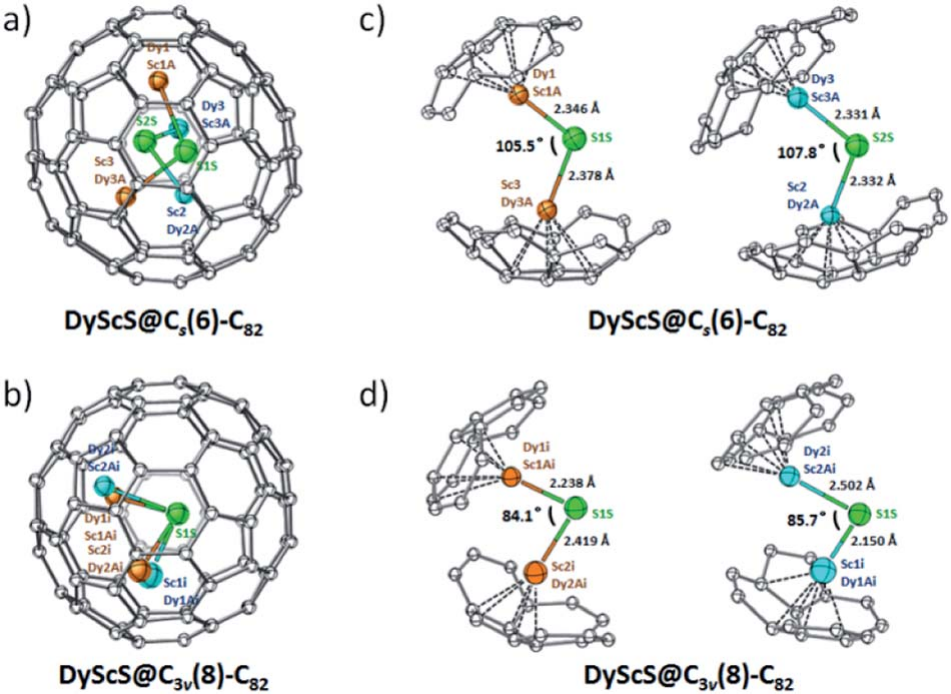
Perspective drawings show (a) the predominant sites of the DyScS cluster within the *C*_s_(6)–C_82_ cage; (b) the predominant sites of the DyScS cluster within the *C*_3v_(8)–C_82_ cage; (c) relative positions of two predominant sites of the DyScS cluster in DyScS@*C*_s_(6)–C_82_; (d) relative positions of two predominant sites of the DyScS cluster in DyScS@*C*_3v_(8)–C_82_. The DyScS unit is modeled with the major site shown in orange and the second major site shown in blue. The metal atoms labeled with ‘i’ are generated by the crystallographic operation.

For DyScS@*C*_s_(6)–C_82_, there are a total of eight Dy/Sc and two sulfide sites in the asymmetric unit. The disordered positions of the DyScS cluster in DyScS@*C*_s_(6)–C_82_ are shown in Fig. S7 (ESI†). As shown in Fig. 3a, the major orientation of the DyScS cluster, which is modeled with Sc3/Dy3A (with fractional occupancy of 0.70), Dy1/Sc1A (with fractional occupancy of 0.46) and S1S (with fractional occupancy of 0.69) according to their occupancies, is highlighted in orange. The second major orientation, which is shown in blue, is modeled with Sc2/Dy2A (with fractional occupancy of 0.24), Dy3/Sc3A (with fractional occupancy of 0.38) and S2S (with fractional occupancy of 0.31) (Fig. 3a). Note that both orientations of the DyScS cluster in DyScS@*C*_s_(6)–C_82_ are analogous to that of the Sc_2_S cluster in Sc_2_S@*C*_s_(6)–C_82_.^26^

For DyScS@*C*_3v_(8)–C_82_, there are fourteen sites for the Dy/Sc atom (including the metal positions generated by the crystallographic mirror plane). Two sites are found for the sulfide atom, and both of them reside on the crystallographic mirror plane. The multiple positions for Dy/Sc atoms indicate that the DyScS cluster tends to move more freely in the *C*_3v_(8)–C_82_ cage compared to the motion in the *C*_s_(6)–C_82_ cage, because the cage carbon signals are averaged to give an apparent *C*_3v_ symmetry to the fullerene. Similar internal dynamic behavior for Y_2_S,^23^ Sc_2_S,^26^ Sc_2_O,^30^ M_2_ and M_2_C_2_ (M = Sc, Y, Lu)^31–34^ were also reported inside the *C*_3v_(8)–C_82_ cage previously.

The disordered positions of the DyScS cluster in DyScS@*C*_3v_(8)–C_82_ are shown in Fig. S8 (ESI†). The major orientation of the DyScS cluster, which is modeled with Sc2i/Dy2Ai (with fractional occupancy of 0.18), Dy1i/Sc1Ai (with fractional occupancy of 0.28) and S1S (with fractional occupancy of 0.34), is highlighted in orange, as shown in Fig. 3b. This configuration is analogous to the major site of the Sc_2_S cluster in Sc_2_S@*C*_3v_(8)–C_82_ as well as the major site of the Dy_2_S cluster in Dy_2_S@*C*_3v_(8)–C_82_.^10,26^ It's worth noting that the Sc1i/Dy1Ai site (with fractional occupancy of 0.14) shows almost identical occupancy with respect to Sc2i/Dy2Ai (with fractional occupancy of 0.18). Thus, it's reasonable to assign the DyScS cluster involving Sc1i/Dy1Ai (with fractional occupancy of 0.14), Dy2i/Sc2Ai (with fractional occupancy of 0.14) and S1S (with fractional occupancy of 0.34) to be the second major orientation considering their occupancies and the bonding distances (Fig. 3b). Both orientations share a common sulfide. This result is in agreement with the previous study that showed that two major crystallographic Dy_2_X sites exist for Dy_2_X@*C*_3v_(8)–C_82_ (X = S, O).^9,10^ DFT calculations for Dy_2_O@*C*_3v_(8)–C_82_ also confirmed that the two most stable conformers are almost isoenergetic within 0.2 kJ mol^−1^.^9^

Fig. 3c and d show the predominant configurations of the DyScS cluster in DyScS@*C*_s_(6)–C_82_ and DyScS@*C*_3v_(8)–C_82_, respectively. Interestingly, the Dy–S–Sc angle varies from 105.5°/107.8° in DyScS@*C*_s_(6)–C_82_ to 84.1°/85.7° in DyScS@*C*_3v_(8)–C_82_. In other words, the DyScS cluster is much less compressed in DyScS@*C*_s_(6)–C_82_ than in DyScS@*C*_3v_(8)–C_82_. Different cluster shapes within isomeric cages were also reported for other Sc-based cluster fullerenes.^30,35^ For example, the Sc–S–Sc angle for Sc_2_S@*C*_s_(6)–C_82_ (113.8°) is considerably larger than that of Sc_2_S@*C*_3v_(8)–C_82_ (97.3°).^26^ Likewise, the Sc–O–Sc angle for Sc_2_O@*C*_s_(6)–C_82_ (156.6°) is also larger than that reported for Sc_2_O@*C*_3v_(8)–C_82_ (131°) (see Table S1, ESI†).^30,35^ Undoubtedly, the cage structure plays an important role on the endohedral cluster shape. However, if a cluster contains larger metal ions, the dimetallic cluster shape in different cage isomers is much less flexible. For example, the Dy_2_S cluster exhibits almost identical Dy–S bond lengths and cluster geometry in Dy_2_S@*C*_s_(6)–C_82_ (98.3°) and in Dy_2_S@*C*_3v_(8)–C_82_ (94.4°).^30^ Similarly, the Dy–O–Dy angle of the major cluster in Dy_2_O@*C*_s_(6)–C_82_ (138.8°) is also comparable to that in Dy_2_O@*C*_3v_(8)–C_82_ (139°) (see Table S1, ESI†).^9^ Thus, cage structures and metal ion sizes exert critical effects on the conformations of the clusters. Moreover, the electrochemical results also confirmed that replacement of the metal in the metal cluster exerts a noticeable influence on their electrochemical behavior (Fig. S9 and Table S2, ESI†).

**Fig. 4:**
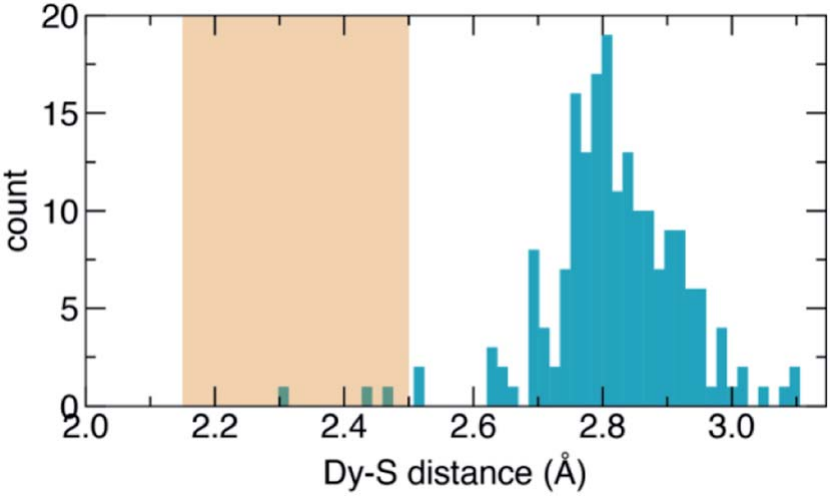
Histogram of Dy–S bond lengths reported in the Cambridge Structural Database (CSD). The orange rectangle shows the range of Dy–S bond lengths observed in the two isomers of DyScS@C_82_.

These crystal cluster geometries are promising for SMM properties. In each of the major and minor clusters, the Dy atoms are coordinated by sulfur on one side and 5- or 6-membered carbon rings from the fullerene cage on the other side, resulting in an axial ligand field. Of particular interest are the short metal–sulfur bond lengths of 2.15 Å to 2.50 Å, which is expected to result in a strong axial field and therefore large single-ion anisotropy, as has been seen in other Dy-based EMF SMMs.^9^ For comparison, we searched the Cambridge Structural Database^36^ for any molecules reporting Dy–S bonds (57 structures with 194 Dy–S bonds) and plotted the bond distances as a histogram (Fig. 4). The median bond length is found to be 2.82 Å, and nearly all lengths are greater than 2.60 Å, significantly larger than Dy–S bonds in DyScS@C_82_. The only exceptions are isomers of Dy_2_S@C_82_,^10^ with Dy–S lengths between 2.44 Å and 2.52 Å, and a coordination polymer with a Dy–S length of 2.298 Å.^37^ From this analysis, it is evident that the fullerene cage in DyScS@C_82_ stabilizes exceptionally short Dy–S bonds. A similar analysis was recently performed for Dy_2_O@C_82_, where the short Dy–O bond lengths stabilized by the fullerene cage were found to result in a very large anisotropy barrier (predicted to be on the order of 1400 cm^−1^) and good SMM performance.^9^ Furthermore, a computational study of the hypothetical EMFs DyScO@C_72–82_, which are oxide analogues of the presently studied EMFs, concluded that the predicted local environments of Dy coordinated by oxygen and carbon, which are similar to the local environments we observe in DyScS@C_82_, are of sufficiently high symmetry to quench QTM up to the third excited states.^19^ Therefore, both isomers of DyScS@C_82_ appear to possess atomic structures well-suited for establishing large thermal barriers to relaxation while also suppressing QTM.

### SMM properties for DyScS@*C*_s_(6)–C_82_ and DyScS@*C*_3v_(8)–C_82_

**Fig. 5:**
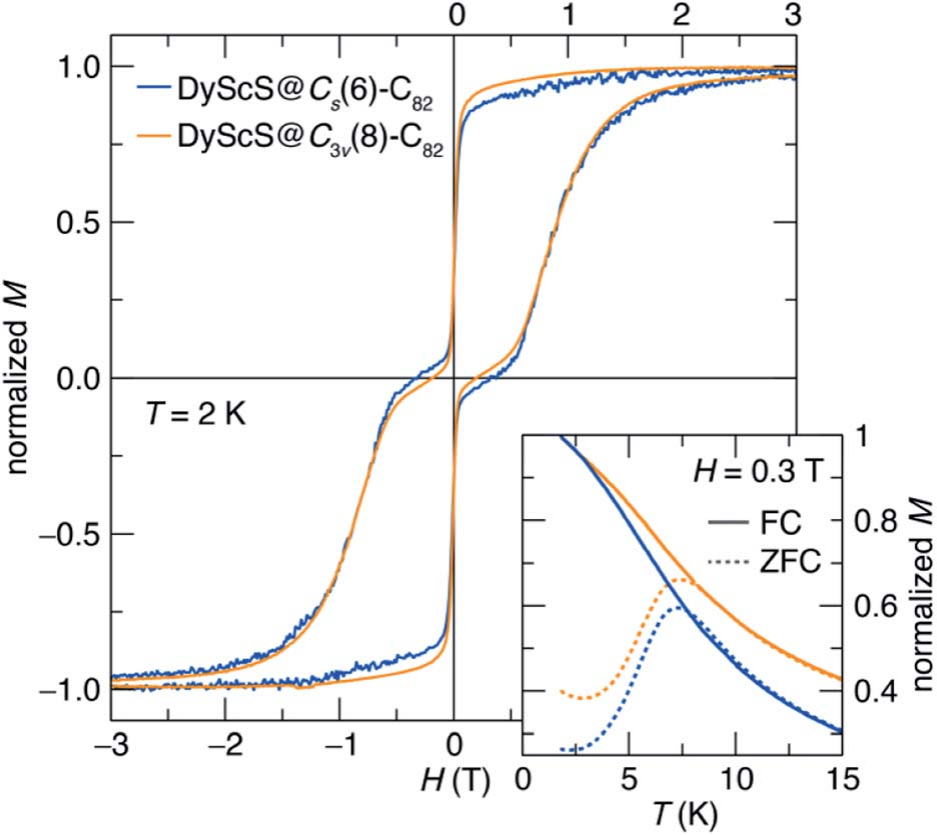
Both isomers of DyScS@C_82_ show magnetic hysteresis and irreversibility at low temperatures, consistent with single-molecule magnet behavior. The main panel shows magnetic hysteresis loops taken at 2 K with a slow field sweep rate of 2.5 mT s^−1^. The inset shows magnetization *vs.* temperature under zero-field cooled (ZFC) and field-cooled (FC) conditions, in each case taken upon warming at a rate of 5 K min^−1^ under an applied field of 0.3 T.

The SMM properties for DyScS@*C*_s_(6)–C_82_ and DyScS@*C*_3v_(8)–C_82_, which were purified on a Buckyprep column, were investigated by means of DC magnetic measurements. Both isomers of DyScS@C_82_ show slow magnetic behavior at low temperature. Fig. 5 shows the magnetic hysteresis loops taken while slowly ramping the field (2.5 mT s^−1^) at 2 K. Both compounds show broad, open loops with a typical “waist-restricted” shape that is characteristic of many SMMs. In these compounds, the widest hysteresis is seen at moderate magnetic fields, where quantum tunneling relaxation is suppressed and magnetic relaxation is at its slowest. Near *H* = 0 T, the loops narrow considerably as quantum tunneling becomes active and increases the rate of relaxation. In many compounds with particularly prominent tunneling relaxation, the waist restriction is so severe that the loop is pinched to a point at *H* = 0 T, for example in HoSc_2_N@C_80_.^38^ On the other hand, in many of the dilanthanide EMF SMMs, where ferromagnetic exchange suppresses quantum tunneling, the waist restriction is considerably reduced or even eliminated.^4,9,39^ Both isomers of DyScS@C_82_ sit in between these two extremes, with a moderate degree of waist-restriction, suggesting that the quantum tunneling is partially suppressed.

While the two isomers show very similar hysteresis behavior, subtle differences may be seen. In particular, DyScS@*C*_3v_(8)–C_82_ shows slightly broader hysteresis than DyScS@*C*_s_(6)–C_82_ under an applied magnetic field, but also shows slightly more waist-restriction near zero field. This suggests that, under a magnetic field, DyScS@*C*_3v_(8)–C_82_ can be expected to have a longer magnetic lifetime than DyScS@*C*_s_(6)–C_82_, while that trend will be reversed at zero field. As with all SMMs, the shape and width of the hysteresis loop are strongly dependent on the magnetic field sweep rate, with substantially more hysteresis seen when the field is swept faster (Fig. S10, ESI†).

Consistent with the magnetic hysteresis at low temperature, both isomers also show irreversibility in magnetization *vs.* temperature measurements taken under zero-field-cooled (ZFC) and field-cooled (FC) conditions at a temperature sweep rate of 5 K min^−1^ (Fig. 5, inset). The magnetic blocking temperature (*T*_B_), defined as the peak temperature in the ZFC curve, is found to be virtually identical for the two isomers (7.33 K for DyScS@*C*_s_(6)–C_82_ and 7.34 K for DyScS@*C*_3v_(8)–C_82_). Interestingly, these values are among the highest blocking temperatures reported for lanthanide-nonmetal endohedral clusters (Table S3, ESI†). Most notably, DyScS@C_82_ shows much higher blocking temperatures than its di-lanthanide analogues Dy_2_S@C_82_ (*T*_B_ ∼ 2 K to 4 K).^10^ This result is in contrast to the dysprosium nitride clusters, where isomers of Dy_2_ScN@C_82_ outperform the monolanthanides DySc_2_N@C_82_.

**Fig. 6:**
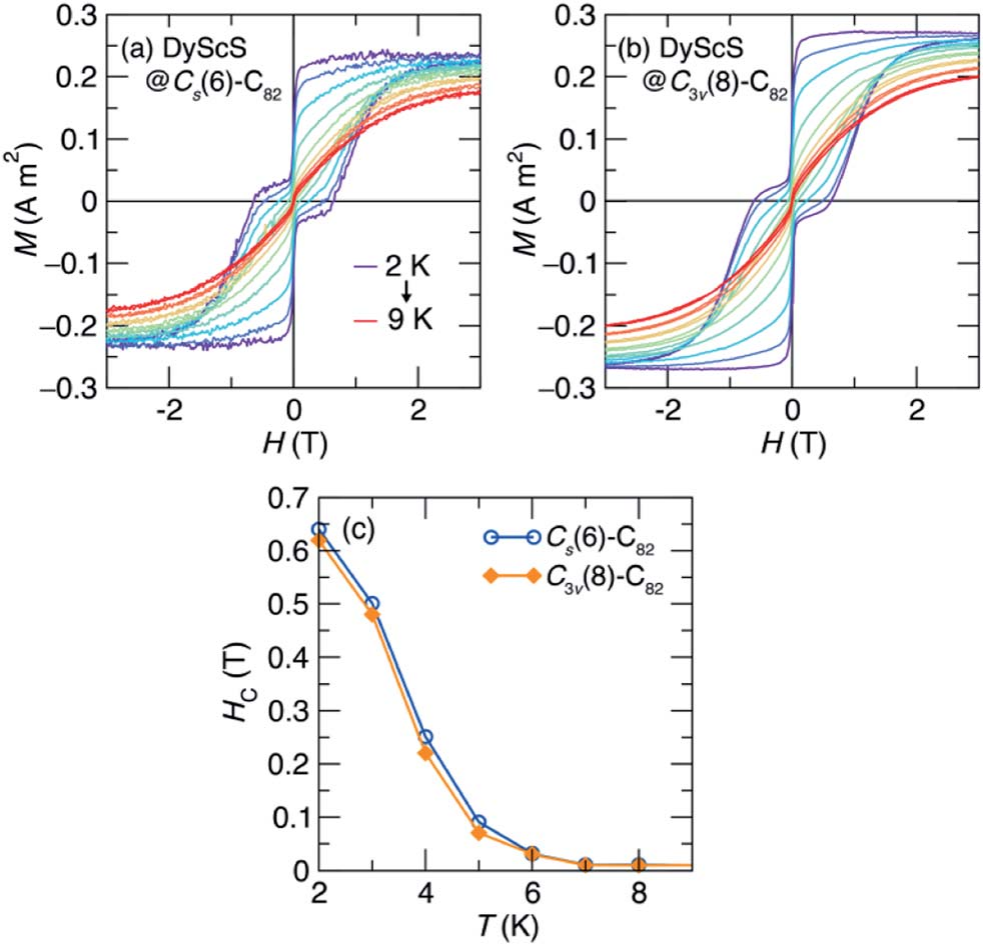
Temperature-dependence of the magnetic hysteresis for (a) DyScS@*C*_s_(6)–C_82_ and (b) DyScS@*C*_3v_(8)–C_82_, collected with a field sweep rate of 10 mT s^−1^. (c) The coercive field (*H*_C_) for each isomer as a function of temperature.

To further explore the blocking behavior, we collected hysteresis loops as a function of temperature, shown in Fig. 6. As temperature is increased from 2 K, the loops narrow and the saturated moment decreases (Fig. 6a and b). At the field-sweep rate used for this experiment (10 mT s^−1^), magnetic hysteresis is still observed until around *T* = 7 K, as can be seen in a plot of the coercive field *vs.* temperature (Fig. 6c).

**Fig. 7:**
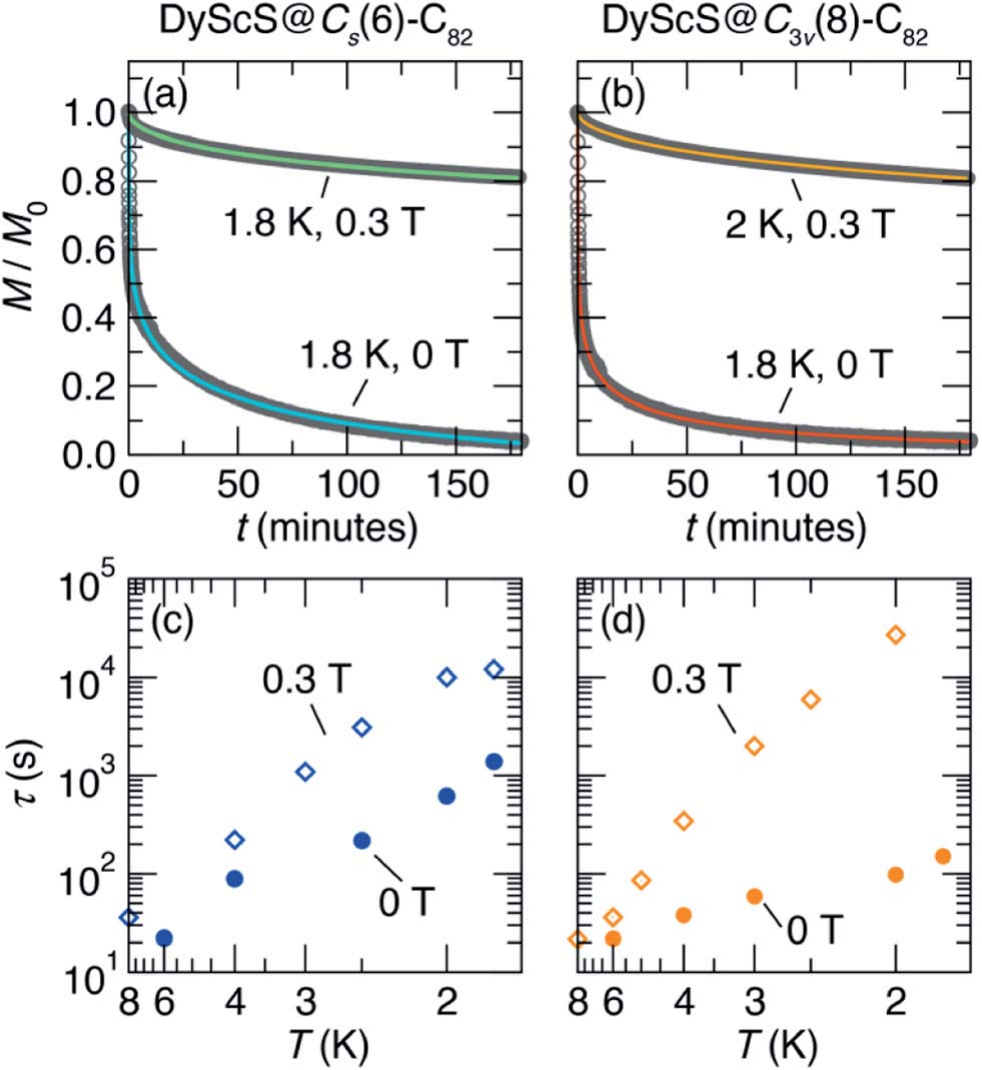
Characterization of magnetic relaxation times of DyScS@*C*_s_(6)–C_82_ (left panels) and DyScS@*C*_3v_(8)–C_82_ (right panels) *via* DC magnetometry. (a) and (b) show representative magnetic relaxation experiments, where the magnetization *M* is monitored as a function of time *t* after the application and subsequent ramp down of a 5 T magnetic field to a target field of either 0 T or 0.3 T. The colored lines indicate fits to stretched exponential functions, which are used to extract the relaxation times. (c) and (d) show the relaxation times extracted using these curves at temperatures ranging from 8 K to 1.8 K, and for applied fields of 0 T and 0.3 T.

Having established the presence of single-molecule magnetism in both isomers of DyScS@C_82_, we then turned to measurements of the magnetic relaxation dynamics. Characteristic magnetic relaxation times as a function of temperature are typically collected using frequency-dependent AC magnetic susceptibility measurements. Unfortunately, we were unable to obtain sufficient AC susceptibility signal given the small amounts of sample isolated, as is frequently the case with EMF SMMs. This means that the sub-second magnetic relaxation dynamics expected at relatively high temperatures are inaccessible. However, below ∼8 K, the magnetic relaxation is slow enough to be probed using DC saturation–relaxation experiments, as shown in Fig. 7. In these experiments, a field of 5 T is applied to the sample at a fixed temperature, and then rapidly ramped down to either 0 T or 0.3 T. Once the target field is hit, the DC magnetization is monitored as a function of time. The resulting decay in magnetization is fit to a model to extract a magnetic lifetime for the given temperature and magnetic field. The decay curves we collected at temperatures between 1.8 K and 2 K are well-fit using a stretched exponential decay function, yielding the relaxation time *vs.* temperature data presented in the Arrhenius plots (log(*τ*) *vs.* 1/*T*) in Fig. 7c and d. Additional details of the fits are provided in the ESI Fig. S11–S15 and Tables S4–S7.†

Based on the waist-restricted shapes of the magnetic hysteresis loops, the application of a moderate magnetic field is expected to suppress quantum tunneling relaxation and therefore result in longer magnetic lifetimes. Indeed, this behavior is observed for both isomers of DyScS@C_82_, with low-temperature lifetimes on the order of minutes for the zero-field data, and hours for the 0.3 T data. Interestingly, this effect is more pronounced for DyScS@*C*_s_(6)–C_82_ than for DyScS@*C*_3v_(8)–C_82_, as predicted by the former's greater degree of waist-restriction seen in the hysteresis loops. At zero field, DyScS@*C*_3v_(8)–C_82_ shows faster relaxation than DyScS@*C*_s_(6)–C_82_ by about an order of magnitude; under a 0.3 T field, however, the trend is reversed.

Typically, thermally activated relaxation in SMMs are fit with the Orbach equation, according to:1
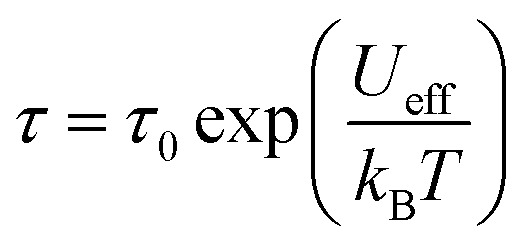
where *U*_eff_ is the effective energy barrier to thermal relaxation, *τ*_0_^−1^ is the attempt frequency, and *k*_B_*T* is the Boltzmann constant times the temperature. However, this linear relation, in general, only fits at high temperatures where other relaxation mechanisms are not active. Without high-temperature relaxation data from AC susceptibility measurements, it is difficult to draw definite conclusions about the mechanisms for the observed magnetic relaxations. In these compounds, the thermal Orbach barrier to relaxation based on the magnetic anisotropy would be expected to be on the order of several hundred K based on results from other Dy-based EMFs.^4,10,40^ However, these barriers cannot be fit with the present data. QTM relaxation is in principle temperature-independent, and therefore at 0 T it would be expected to plateau as the temperature is decreased. In our 0T data, however, a definite temperature-dependence of the relaxation times is observed down to the lowest temperature measured. Given that the field-dependence of the relaxation times and the waist-restricted hysteresis clearly indicates the presence of QTM, it is not clear what the origin for this behavior is; however, similar behavior was observed for DySc_2_N@C_80_ and was tentatively explained as a phonon bottleneck to energy dissipation.^8^ Attempts to fit our low-temperature data to Orbach processes yielded barriers between 3 K to 14 K and *τ*_0_ between 2 s and 11 s (Fig. S16 and Table S8, ESI†).

Once again, it is of interest to compare the SMM performance of DyScS@C_82_ to the performance of Dy_2_S@C_82_ isomers, which were reported to display non-waist-restricted hysteresis loop consistent with suppressed quantum tunneling of magnetization due to Dy–Dy ferromagnetic exchange.^10^ Interestingly, even though DyScS@C_82_ does not display such a complete suppression of tunneling, its magnetic relaxation times far exceed those of Dy_2_S@C_82_, even at zero field. At 1.8 K, the *C*_s_ and *C*_3v_ isomers of Dy_2_S@C_82_ show zero-field magnetic lifetimes around 10 s and 100 s, respectively.^10^ For DyScS@C_82_, these lifetimes are 1390(40) s and 150(1) s, respectively. Application of a 0.3 T magnetic field further increases these values up to 1.202(1) × 10^4^ s and ∼5 × 10^4^ s, respectively. For the same isomers of Dy_2_O@C_82_, the lifetimes are long at 1.8 K in zero field (10^3^ s to 10^4^ s), but are suppressed by the application of moderate magnetic fields.^9^

The difference in behavior between DyScS@C_82_ and Dy_2_S@C_82_ may be rationalized on the basis of the Dy–Dy exchange interaction in Dy_2_S@C_82_. Even though the ferromagnetic Dy–Dy interaction seems to suppress quantum tunneling, the overall observed relaxation times still plateau as the temperature is decreased. This behavior can be explained by the fact that the exchange interaction is weak in di-lanthanide clusters, leading to low-lying excited exchange states. As a result, low-energy Orbach processes, with barriers of 15.2 K (*C*_s_(6)–C_82_) and 6.5 K (*C*_3v_(8)–C_82_), dominate the relaxation at low temperatures in Dy_2_S@C_82_.^10^ DyScS@C_82_ has no such ferromagnetic exchange. Therefore, QTM is not as completely suppressed, but the exchange relaxation pathway is not available. Therefore, changing from mono-lanthanide to di-lanthanide clusters represents a tradeoff. In some Dy-based EMFs, the tradeoff of exchange relaxation for QTM suppression results in better performance for the dilanthanide.^40^ In the Dy sulfide clusters, however, the monolanthanides evidently far outperform the dilanthanides.

## Conclusions

In this work, two new dysprosium-containing mixed dimetallic sulfide clusterfullerenes, namely, DyScS@C_82_ (I, II), have been successfully synthesized and characterized by mass spectrometry, Vis-NIR, cyclic voltammetry, single crystal X-ray diffractometry, and magnetic measurements. Crystallographic analyses revealed that DyScS@C_82_ (I, II) possess *C*_s_(6)–C_82_ and *C*_3v_(8)–C_82_ cages, respectively. Notably, the metal ion size of the cluster exhibits a critical effect on the conformation of the cluster in the fullerene cages. Results from redox potentials also show that replacement of the metal in the metal cluster exerts a noticeable influence on their electrochemical behavior.

Both isomers of DyScS@C_82_ show very similar single molecule magnetic behavior with open hysteresis loops at low temperature. The magnetic blocking temperatures are both around 7.3 K, among the highest reported values for clusterfullerene SMMs. This promising behavior is attributed to the strong axial field generated by short Dy–S distances. Notably, the SMM blocking temperatures and magnetic lifetimes far exceed those for the dimetallic sulfide EMF, Dy_2_S@C_82_. This result underlines the promise of ECFs with single Dy atoms and short metal–nonmetal contacts. Therefore, the (so far unreported) compound DyScO@C_82_ may be expected to perform very well as a SMM. Furthermore, a recent report has shown that the identity of the diamagnetic metal in a Dy cluster can have a large impact on the SMM properties,^19^ so the full series of compounds DyMX@C_82_ (M = Y, Sc, Lu; X = O, S) represents a fruitful research direction.

## Conflicts of interest

There are no conflicts to declare.

## Supplementary Material

SC-011-D0SC05265E-s001

SC-011-D0SC05265E-s002
