# Supplementary material for: High blocking temperatures for DyScS endohedral fullerene single-molecule magnets
Source: Chem Sci. 2020 Nov 2;11(48):13129–36. doi: 10.1039/d0sc05265e (PMC8163201; doi:10.1039/d0sc05265e)
Supplement: SC-011-D0SC05265E-s001 [file SC-011-D0SC05265E-s001.pdf]

Electronic Supplementary Material (ESI) for Chemical Science.
This journal is © The Royal Society of Chemistry 2020

Supporting Information of

High blocking temperatures for DyScS endohedral fullerene single-molecule magnets

Wenting Cai,[a] Joshua D. Bocarsly,[b] Ashley Gomez,[a] Rony J Letona Lee,[a] Alejandro Metta-Magaña,[a] Ram Seshadri,[b] Luis Echegoyen*,[a]

[a] Department of Chemistry, University of Texas at El Paso, 500 W University Avenue, El Paso, Texas 79968 (United States)

[b] Materials Research Lab and Materials Department, University of California Santa Barbara, California 93106 (United States)

Table of Contents

1. Synthesis of DyScS@C_{82}..S2
2. HPLC separation of DyScS@C_s(6)-C_{82} and DyScS@C_{3v}(8)-C_{82}.........................S2
3. Single crystal X-ray diffraction studies details……………………………………..S6
4. Electrochemical Studies……………………………………………………………S7
5. Magnetic measurements details……………………….................................S10
6. References…………………………………………………………………..…S16

1. Synthesis of DyScS@C_{82}. The carbon soots containing DyScS@C_{82} were synthesized by the direct-current arc discharge method. The graphite rods, packed with Dy_2O_3/Sc_2O_3/graphite powder in a weight ratio of 2.6:1:3, were vaporized in the arcing chamber under 210 torr He and 20 torr SO_2. The resulting soot was refluxed in CS_2 under an argon atmosphere for 12 h. DyScS@C_{82} and DyScS@C_{84} were obtained along with a family of Sc_2S@C_{2n} (2n = 82-90) (Figure S1).

2. HPLC separation of DyScS@C_s(6)-C_{82} and DyScS@C_{3v}(8)-C_{82}. The separation and purification of DyScS@C_{82}(I, II) were achieved by multistage HPLC procedures. The first stage HPLC separation was performed on a 5PYE column (10 mm x 250mm, Cosmosil Nacalai Tesque) with toluene as the eluent. Figure S2 shows the first stage HPLC chromatogram of extract sample. Two fractions were collected, A and B, respectively; both containing DyScS@C_{82}. After that, fraction A was injected into a Buckyprep column (10 mm x 250mm, Cosmosil Nacalai Tesque) for the second stage separation with toluene as the eluent (Figure S3a). Fraction A1 was collected and then injected into a Buckyprep-M column (10 mm x 250mm, Cosmosil Nacalai Tesque) with a toluene mobile phase. Fraction A1-1 containing both Sc_2S@C_{82} and DyScS@C_{82} was then collected (Figure S3b). In order to remove Sc_2S@C_{82}, the fourth stage separation for A1-1 was carried out using a 5PBB column (4.9 mm x 250mm, Cosmosil Nacalai Tesque) with a toluene mobile phase, in which pure DyScS@C_{82}(I) was obtained (Figure S3c). In addition, fraction B was also injected into a Buckyprep column (10 mm x 250mm, Cosmosil Nacalai Tesque) for the second stage separation with toluene as the eluent (Figure S4a). Fraction B1 was collected and re-injected into a Buckyprep-M column (10 mm x 250mm, Cosmosil Nacalai Tesque) with a toluene mobile phase. Fraction B1-1 containing Sc_2S@C_{82} and DyScS@C_{82} was then collected (Figure S4b). Similarly, the final step separation was conducted on a 5PBB column (4.9 mm x 250mm, Cosmosil Nacalai Tesque) with a toluene mobile phase to obtain pure DyScS@C_{82}(II) (Figure S4c). The purity of the isolated DyScS@C_{82} (I, II) were confirmed by the single peak on the final-stage HPLC chromatograms and MALDI-TOF mass spectrometry (Figure S5).

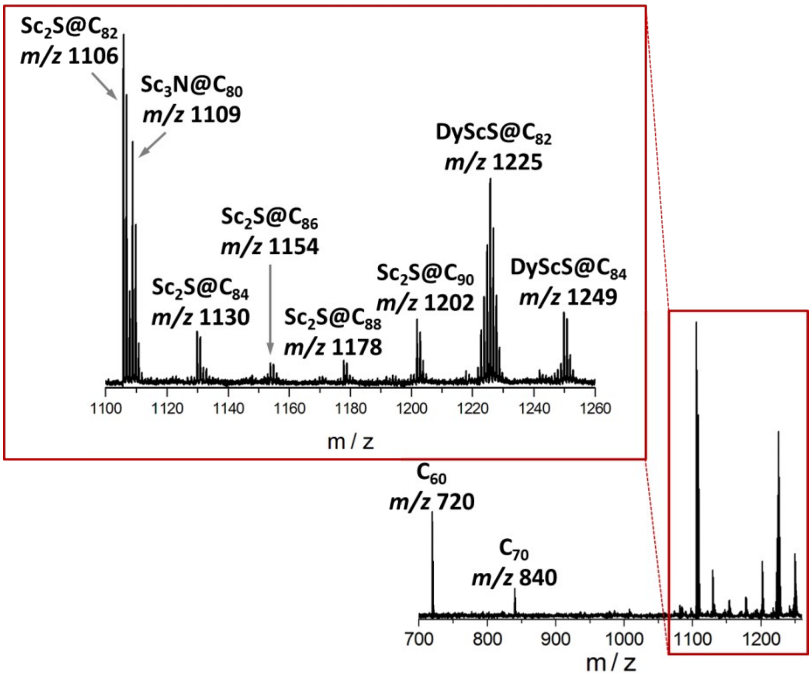

Figure S1. MALDI-TOF spectra of the extract showing the existence of $DyScS@C_{2n}$ (2n = 82, 84) and a family of $Sc_2S@C_{2n}$ (2n = 82-90).

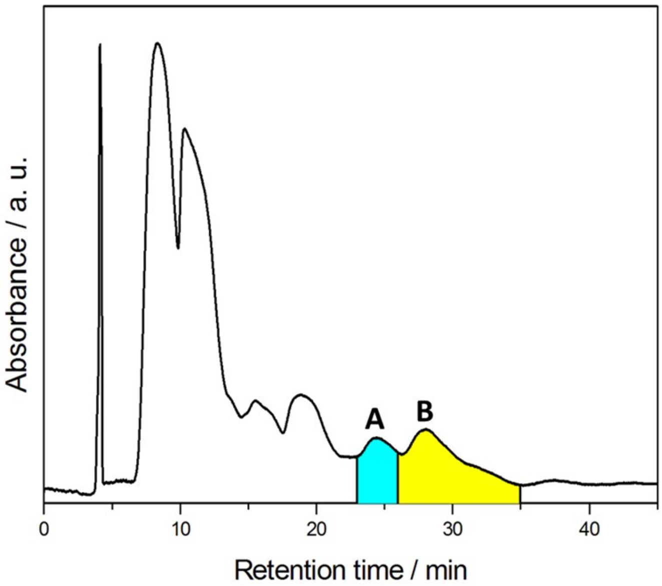

Figure S2. The first stage HPLC chromatogram of extract. HPLC conditions: 5PYE column, Φ = 10 mm x 250 mm; eluent = toluene; flow rate = 4 mL/min; detecting wavelength = 390nm.

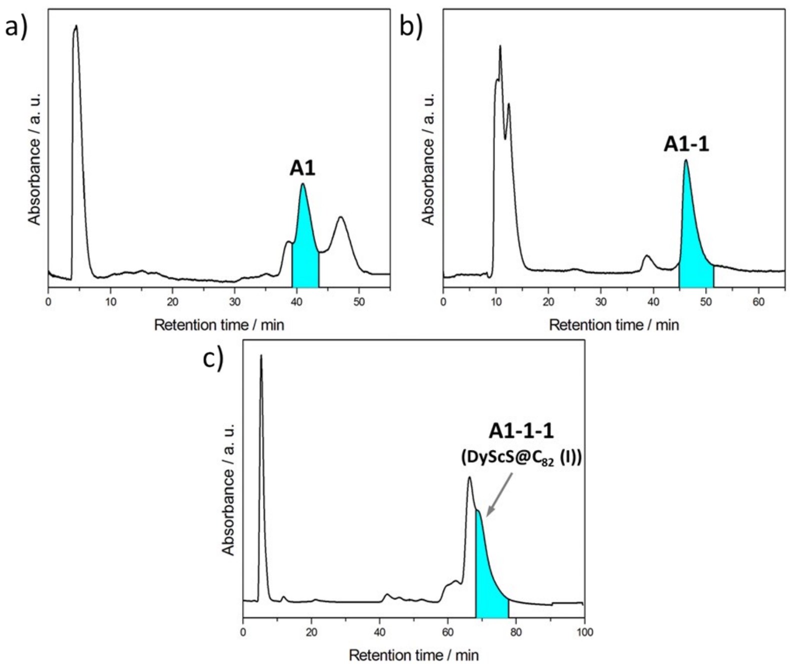

Figure S3. HPLC separation of DyScS@C_{82} (I). (a) The second stage HPLC chromatogram of fraction A on a Buckyprep column (Φ = 10 mm × 250 mm). The HPLC conditions was: eluent = toluene; flow rate = 4 mL/min; detecting wavelength = 390 nm. (b) The third stage HPLC chromatogram of fraction A1 on a Buckyprep-M column (Φ = 10 mm × 250 mm). The HPLC conditions was: eluent = toluene; flow rate = 2 mL/min; detecting wavelength = 390 nm. (c) The fourth stage HPLC chromatogram of fraction A1-1 on a 5PBB column (Φ = 4.9 mm × 250 mm). The HPLC conditions was: eluent = toluene; flow rate = 1 mL/min; detecting wavelength = 390 nm.

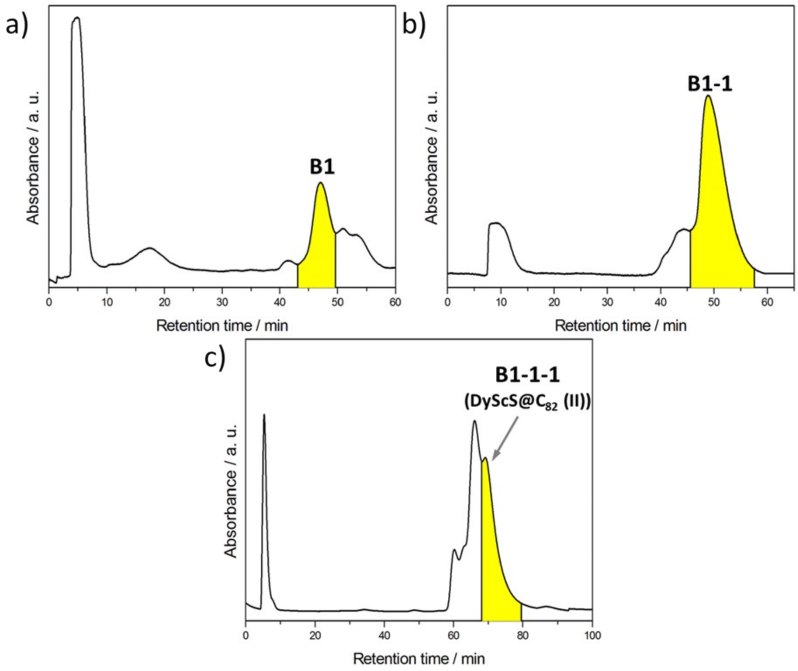

Figure S4. HPLC separation of DyScS@C_{82} (I). (a) The second stage HPLC chromatogram of fraction B on a Buckyprep column (Φ = 10 mm × 250 mm). The HPLC conditions was: eluent =

toluene; flow rate = 4 mL/min; detecting wavelength = 390 nm. (b) The third stage HPLC chromatogram of fraction B1 on a Buckyprep-M column (Φ = 10 mm × 250 mm). The HPLC conditions was: eluent = toluene; flow rate = 2 mL/min; detecting wavelength = 390 nm. (c) The fourth stage HPLC chromatogram of fraction B1-1 on a 5PBB column (Φ = 4.9 mm × 250 mm). The HPLC conditions was: eluent = toluene; flow rate = 1 mL/min; detecting wavelength = 390 nm.

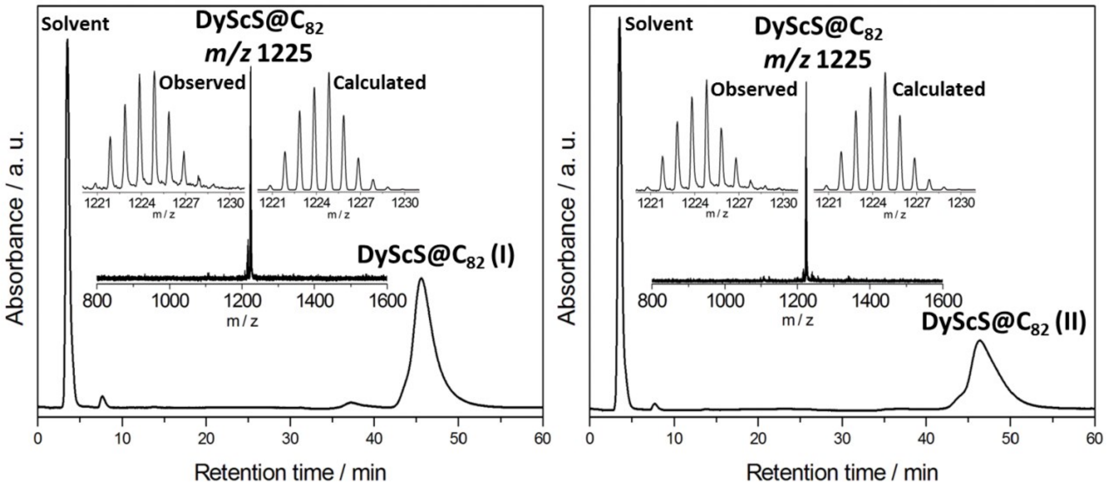

Figure S5. HPLC chromatograms of purified DySc$S@C_{82}$ (I, II) on a 5PBB column (Φ = 4.9 mm × 250 mm) with toluene as the eluent at the flow rate of 1.5 mL·min^{-1}; Insets show the positive mode MALDI-TOF mass spectra and expansions of the experimental and theoretical isotopic distributions of DySc$S@C_{82}$ (I, II).

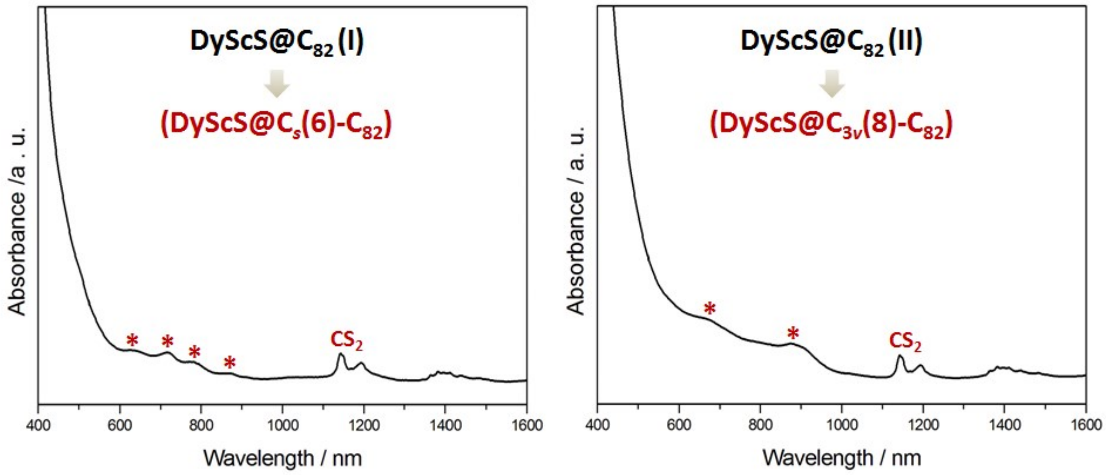

Figure S6. Vis-NIR absorption spectra of DySc$S@C_{82}$ (I, II) in CS_2.

3. Single crystal X-ray diffraction studies details. Crystalline blocks of DyScS@C_{82} were obtained by layering a benzene solution of Ni^{II}(OEP) over a nearly saturated solution of the respective endohedral in CS_2 in a glass tube. Over a 20-day period, the two solutions diffused into each other and black crystals formed. XRD measurements were performed at 150 K on a Bruker APEX-II CCD diffractometer. The multi-scan method was used for absorption corrections. The structures were solved by a direct method and were refined with SHELXL-2018.[1] Hydrogen atoms were inserted at calculated positions and constrained with isotropic thermal parameters. All metallic sites are treated as overlapped Dy/Sc positions using the combination of EXYZ and EADP commands.

The asymmetric unit for DyScS@C_s(6)-C_{82}·Ni^{II}(OEP)·2C_6H_6 exhibits a fully ordered fullerene cage. However, the asymmetric unit for DyScS@C_{3v}(8)-C_{82}·Ni^{II}(OEP)·CS_2·1.5C_6H_6 contains a symmetry-related Ni^{II}(OEP) molecule and two halves of the C_{3v}(8)-C_{82} cage. The intact cage is generated by combining one-half of the cage with the mirror image of the other, both having an occupancy value of 0.50.

Crystal data for DyScS@C_s(6)-C_{82}·Ni^{II}(OEP)·2C_6H_6: $C_{130}H_{56}DyN_4NiSSc$, M_w = 1972.01, monoclinic, space group $P2_1/c$, a = 19.994(8) Å, b = 15.029(6) Å, c = 25.360(6) Å, β= 94.394(10)°, V = 7598(5) Å^3, Z = 4, T = 150 K, ρ_{calcd} = 1.724 Mg m^{-3}, μ(MoKα) = 1.404 mm^{-1}, 37653 reflections measured, 21747 unique (R_{int} = 0.1476 used in all calculations. The final wR_2 was 0.3659 (all data) and R_1 (8740 with I>2\s(I)) = 0.1637. CCDC 1990729 contains the crystallographic data.

Crystal data for 2DyScS@C_{3v}(8)-C_{82}·2Ni^{II}(OEP)·2CS_2·3C_6H_6: $C_{256}H_{106}Dy_2N_8Ni_2S_6Sc_2$, M_w = 4018.18, monoclinic, space group C_2/m, a = 26.8856(18) Å, b = 17.1624(11) Å, c = 17.8103(12) Å, β= 106.578(3)°, V = 7876.4(9) Å^3, Z = 2, T = 150 K, ρ_{calcd} = 1.694 Mg m^{-3}, μ(MoKα) = 1.407 mm^{-1}, 88881 reflections measured, 12338 unique (R_{int} = 0.0405 used in all calculations. The final wR_2 was 0.3882 (all data) and R_1 (8646 with I>2\s(I)) = 0.1636. CCDC 1990730 contains the crystallographic data.

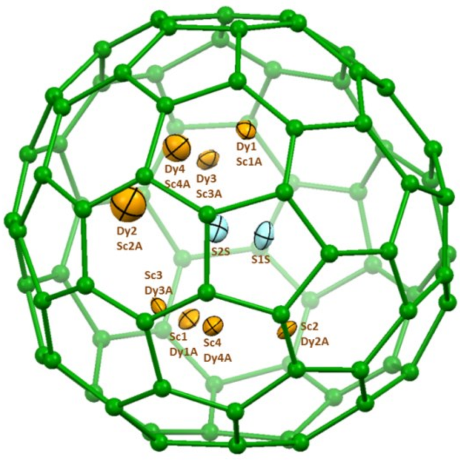

Figure S7. Drawing showing the DyScS disordered positions in DyScS@C_s(6)-C_{82}.

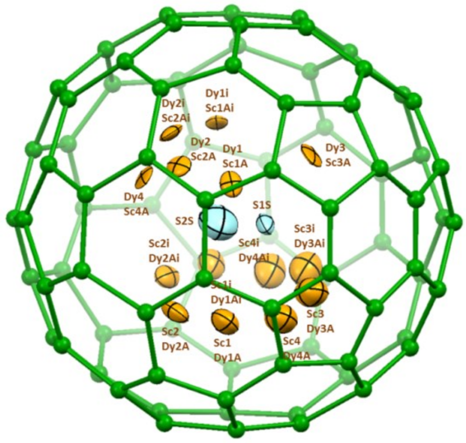

Figure S8. Drawing showing the DyScS disordered positions in DyScS@C_{3v}(8)-C_{82}. The metal atoms labeled with 'i' are generated by the crystallographic operation.

Table S1. Comparison of dihedral angles in selected $Dy_mSc_{2-m}X$@C_s(6)-C_{82} and $Dy_mSc_{2-m}X$@C_{3v}(8)-C_{82} (X = S, O; m = 0-2) from X-ray diffraction.

	DyScS	Sc_2S[2]	Sc_2O[3, 4]	Dy_2S[30]	Dy_2O[5]
C_s(6)-C_{82}	105.5°/107.8°	113.8°/115.2°	156.6°	98.3°	138.8°
C_{3v}(8)-C_{82}	84.1°/85.7°	97.3°	131°	94.4°	139°/145°

4. Electrochemical Studies. The electrochemical properties of DyScS@C_s(6)-C_{82} and DyScS@C_{3v}(8)-C_{82} were investigated by means of cyclic voltammetry (CV) using *ortho*-dichlorobenzene and tetra(*n*-butyl)ammonium hexafluoro-phosphate (*o*-DCB/n-Bu_4NPF_6) as solvent/electrolyte (see Figure S8). A conventional three-

electrode cell consisting of a platinum counter-electrode, a glassy carbon working electrode, and a silver reference electrode was used for all measurements. All potentials were reported relative to the Fc/Fc^+ couple. Two reversible reductive steps and one reversible oxidative step were observed for DyScS@C_s(6)-C_{82}. However, other compounds with the same C_s(6)-C_{82} cage and similar encapsulated clusters, namely, Sc_2S@C_s(6)-C_{82},[2, 6] Sc_2O@C_s(6)-C_{82}[2] and Dy_2O@C_s(6)-C_{82},[5] exhibiting very different electrochemical behavior. Likewise, the electrochemical behavior of DyScS@C_{3v}(8)-C_{82} is not similar to that for Sc_2S@C_{3v}(8)-C_{82},[2, 6] Sc_2O@C_{3v}(8)-C_{82}[4] or Dy_2O@C_{3v}(8)-C_{82}.[5] Even though all of them exhibit two oxidation processes, their reduction processes show very different patterns. Specifically, the cyclic voltammetry of DyScS@C_{3v}(8)-C_{82} shows three reversible and two irreversible reductive steps, whereas Sc_2S@C_{3v}(8)-C_{82}[2, 6] exhibits one irreversible and two reversible reduction peaks and the other two oxide cluster endohedrals (Sc_2O@C_{3v}(8)-C_{82}[4] and Dy_2O@C_{3v}(8)-C_{82}[5]) exhibit four reduction steps.

The observed redox potentials for DyScS@C_s(6)-C_{82}, DyScS@C_{3v}(8)-C_{82}, Sc_2S@C_s(6)-C_{82}[2, 6] and Sc_2S@C_{3v}(8)-C_{82}[2, 6] are summarized in Table S2. For comparison, four oxide cluster endohedral fullerenes, M_2O@C_s(6)-C_{82} and M_2O@C_{3v}(8)-C_{82} (M = Sc, Dy),[2, 4, 5] are also included in the Table because the nonmetal atom in the cluster normally exerts a more negligible contribution on the electrochemical behavior of endohedral fullerenes. Therefore, it was not surprising to see a similarity of the redox potentials between Sc_2S@C_{82}[2, 6] and Sc_2O@C_{82}.[2, 4] However, when one Sc atom is replaced by one Dy atom, the first oxidation potential (0.22 V) and the first reduction potential (-1.06 V) for DyScS@C_s(6)-C_{82} are cathodically shifted relative to those of Sc_2S@C_s(6)-C_{82}[2, 6] and Sc_2O@C_s(6)-C_{82}.[2] For DyScS@C_{3v}(8)-C_{82}, the first oxidation peak (0.18 V) is even more dramatically shifted relative to those of Sc_2S@C_{3v}(8)-C_{82} (0.52 V)[2, 6] and Sc_2O@C_{3v}(8)-C_{82} (0.54 V),[4] and the first reduction potential (-0.85V) is anodically shifted compared to those for Sc_2S@C_{3v}(8)-C_{82} (-1.04 V)[2, 6] and Sc_2O@C_{3v}(8)-C_{82} (-1.17 V).[4] Likewise, when two Sc atoms are replaced by two Dy atoms, the redox potentials of Dy_2O@C_s(6)-C_{82}[5] and Dy_2O@C_{3v}(8)-C_{82}[5] are obviously changed compared with either Sc_2X@C_{82} (X = S, O) isomers[2, 4, 6] or DyScS@C_{82} isomers (see Table S2). These results verified that, even though the HOMO and LUMO for M_2X@C_s(6)-C_{82} and M_2X@C_{3v}(8)-C_{82} (M = Sc, Dy; X = S, O) are mainly delocalized over the fullerene cage with negligible contributions from the cluster,[5, 6] the different metal atoms in the clusters can exert a

strong influence on the electrochemical behavior of endohedral fullerenes possessing isoelectronic clusters and same cage symmetries. It's also interesting to note that the resulting electrochemical gaps for $Sc_2X@C_{82}$ (X = S, O) isomers are always larger than those for the $DyScS@C_{82}$ and $Dy_2O@C_{82}$ isomers, indicating the higher stability of $Sc_2X@C_{82}$ (X = S, O) relative to those for the Dy-containing cluster fullerenes.

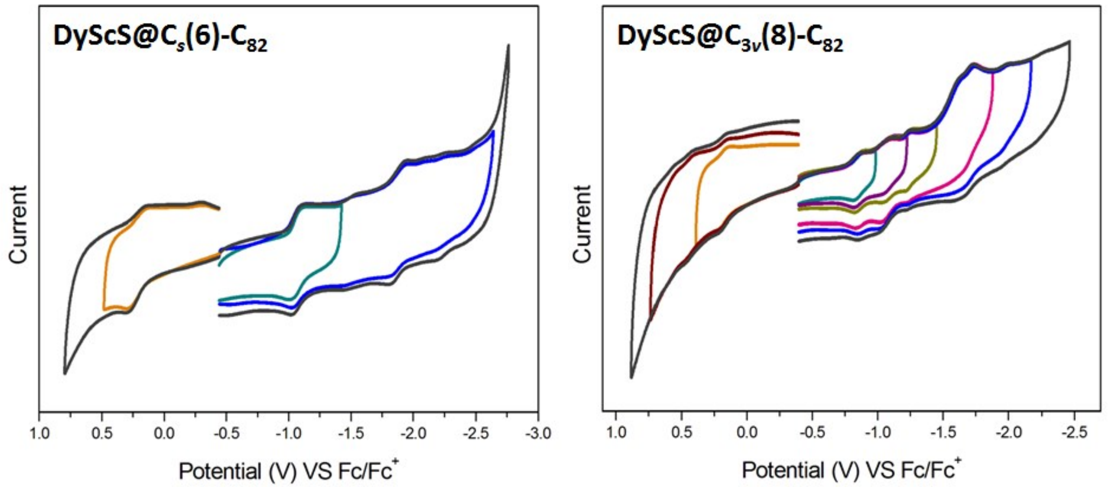

Figure S9. Cyclic voltammograms of DyScS@C_s(6)-C_{82} (left) and DyScS@C_{3v}(8)-C_{82} (right) in *o*-dichlorobenzene (0.05 M (*n*-Bu)$_4$NPF$_6$; scan rate 100 mV/s for CV).

Table S2. Redox potentials (V vs Fc/Fc$^+$) and electrochemical bandgaps of DyScS@C_s(6)-C_{82}, DyScS@C_{3v}(8)-C_{82} and reference endohedrals.

Species	$^{ox}E_3$	$^{ox}E_2$	$^{ox}E_1$	$^{red}E_1$	$^{red}E_2$	$^{red}E_3$	$^{red}E_4$	$^{red}E_5$	ΔE_{gap}
DyScS@C_s(6)-C_{82}	/	/	0.22^a	-1.06^a	-1.89^a	/	/	/	1.28
Sc_2S@C_s(6)-C_{82}[2, 6]	0.98	0.65	0.39	-0.98	-1.12	-1.73	/	/	1.37
Sc_2O@C_s(6)-C_{82}[2]	/	0.72	0.35	-0.96	-1.28	-1.74	/	/	1.31
Dy_2O@C_s(6)-C_{82}[5]	0.95^b	0.42^a	0.19^a	-0.75^a	-1.17^a	-1.86^a	-2.24^a		0.94
DyScS@C_{3v}(8)-C_{82}	/	0.45^a	0.18^a	-0.85^a	-1.09^a	-1.25^a	-1.75^b	-2.01^b	1.03
Sc_2S@C_{3v}(8)-C_{82}[2, 6]	/	0.96	0.52	-1.04	-1.19	-1.63	/	/	1.56
Sc_2O@C_{3v}(8)-C_{82}[4]		1.09^b	0.54^a	-1.17^b	-1.44^b	-1.55^b	-1.78^b		1.71
Dy_2O@C_{3v}(8)-C_{82}[5]		0.91^b	0.43^a	-0.77^b	-1.20^a	-1.78^a	-2.08^a		1.20

^{a} Half-cell potentials are given unless otherwise addressed.

^{b} Irreversible. Square wave voltammetry peak value.

5. Details of the magnetic measurements. DC magnetic measurements were performed using a Quantum Design SQUID magnetometer (MPMS 3) operating in Vibrating Sample Magnetometer (VSM) mode and a Quantum Design Physical Property Measurement System (Dynacool PPMS) outfitted with a VSM. For each isomer, a small amount of sample (<1 mg) was dispersed in CS_2 and cast into a polypropylene sample holder, which was then placed into a brass sample holder for measurement in either device. Due to the small amount of sample available, we were unable to obtain accurate sample masses. Therefore, we are unable to provide measurements of the magnetic moment on a per-molecule basis. However, measurements of magnetic hysteresis, blocking, and magnetic lifetimes were performed. Magnetic hysteresis loops were collected while sweeping the magnetic field at a constant sweep rate, cycling between fields of 5 T and –5T while measuring continuously in VSM mode. The linear diamagnetic signal from the sample holder was subtracted from the measurements. Magnetization *vs.* temperature was measured continuously in VSM mode while warming from 2 K to 40 K at a rate of 5 K min^{-1} under a magnetic field of 0.3 T. These measurements were performed both after cooling in zero magnetic field (ZFC) and after cooling in a 0.3 T magnetic field (FC). The magnetic blocking temperature T_B was taken as the maximum in the ZFC measurement.

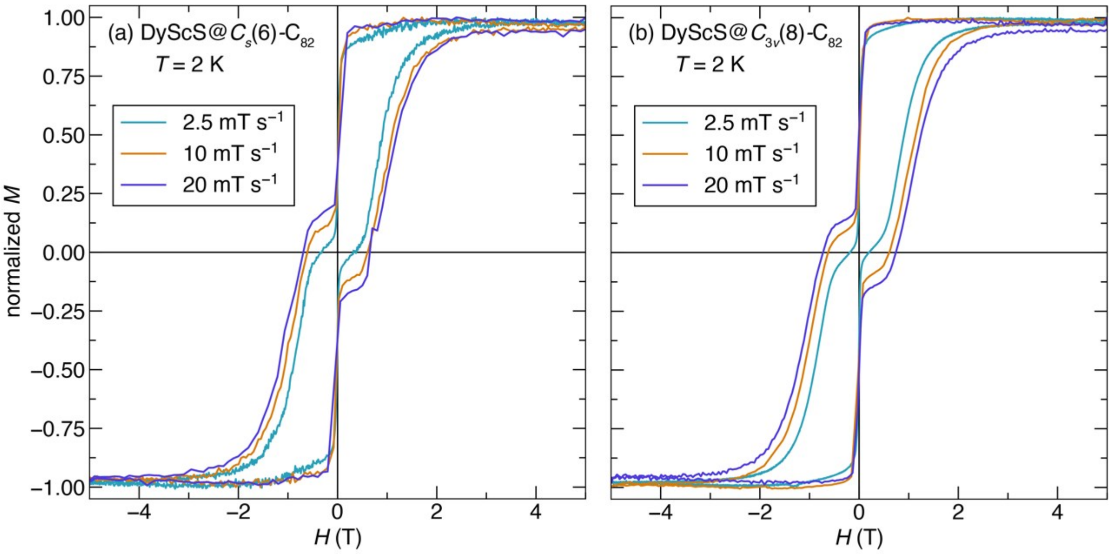

Figure S10. Dependence of the 2 K magnetic hysteresis loops on the field sweep rate. More hysteresis is seen when the field is swept faster, as is expected for single-molecule magnets.

Table S3: Selected blocking temperatures for some Dy-based EMFs

	T_B (K)[a]	$T_{B,100}$ (K)[b]	Ref.
$DyScS@C_s(6)-C_{82}$	7.3	5.4	this work
$DyScS@C_{3v}(8)-C_{82}$	7.3	4.9	this work
$Dy_2S@C_{3v}(8)-C_{82}$	4	2	[7]
$Dy_2S@C_s(6)C_{82}$	~2		[7]
$Dy_2S@C_s(10528)-C_{72}$	< 2		[7]
$Dy_2O@C_s(6)-C_{82}$	4.4	2.8	[5]
$Dy_2O@C_{3v}(8)-C_{82}$	7.4	5.9	[5]
$Dy_2O@C_{2v}(9)-C_{82}$	5.8	3.7	[5]
$Dy_2O@C_{72}$	4	3.4	[8]
$Dy_2O@C_{74}$	6.7	5	[8]
$DySc_2N@D_3(6140)-C_{68}$	3.8	2.3	[9]
$DySc_2N@I_h(7)-C_{80}$	7	4.6	[10, 11]
$DySc_2N@D_{5h}(6)-C_{80}$	5.9	3.6	[9]
$Dy_2ScN@I_h(7)-C_{80}$	8	5	[12, 13]
$Dy_2ScN@D_{5h}(6)-C_{80}$	5.3	2.6	[9]
$Dy_2ScN@C_s(51365)-C_{84}$	3.3	~1.8	[9]
$Dy_2@C_{80}(CH_2Ph)$	21.9	18	[14]

[a] Here, the blocking temperature T_B is defined as the peak temperature of χ_{ZFC} while warming at a rate of 5 K min^{-1}.

[b] The 100 s blocking temperature $T_{B,100}$ is the temperature at which 100 s relaxation time is observed.

Lastly, magnetic saturation-relaxation experiments were performed to obtain magnetic relaxation times of each isomer at several temperatures, both at 0 T and at 0.3 T. In each case, the sample was magnetized to 5 T at a given temperature for five minutes, and then field was then brought down to either 0 T or 0.3 T at a rate of 20-70 mT s^{-1}, and the magnetization as a function of time was recorded. The resulting decay curves were fit to a stretched exponential decay function:

$$\frac{M(t)}{M_0} = (1 - y_0)e^{-\left(\frac{t}{\tau}\right)^b} + y_0\#(S1)$$

where the left side is the magnetization M as a function of time t, normalized by the $t = 0$ magnetization M_0. τ is the relaxation time, b is a positive number between 0 and 1, and y_0 is the normalized magnetization at $t = \infty$. τ, b, and y_0 are the fit parameters.

The results of these fits are given in Tables S4 – S7 and Figures S9, S10, S11, and S13.

For very long relaxation times in a magnetic field, fits to equation S1 can sometimes suffer from high correlations between the fit parameters. To remedy this, the 1.8 K data of isomer 1 was fit using both relaxation data and field application data, as shown in Figure S. The field application data was taken by cooling the sample in zero field, and then applying a 0.3 T and measuring magnetization as a function of time. The eventual saturation magnetization of this process should match the final magnetization of the saturation decay data, so these two processes can be fit together to obtain accurate relaxation times. The field application data was fit to:

$$\frac{M(t)}{M_0} = y_0 - (y_0 - M_0')e^{-\left(\frac{t}{\tau}\right)^b} + y_0 \#(S2)$$

Where M_0 is the initial magnetization of the decay data set (the same value as in equation S1), and M_0' is the initial magnetization of the field application data set. The fit parameters τ, b, and y_0 have the same meanings as in equation S1, and these values are constrained to be equal for the two data sets.

Table S4. Relaxation times for DyScS@C_s(6)-C_{82} at 0 T

T (K)	M_0 **(m-emu)**	τ **(s)**	***b***	y_0
6.0	0.0300	22.11(2)	0.6539(5)	0.02553(1)
4.0	0.0505	88.5(7)	0.548(3)	0.0191(2)
2.5	0.0778	217(1)	0.416(1)	–0.0008(2)
2.0	0.0822	617(5)	0.349(1)	–0.032(1)
1.8	0.0895	1390(40)	0.258(2)	–0.183(5)

Table S5. Relaxation times for DyScS@C_s(6)-C_{82} at 0.3 T

T (K)	M_0 **(m-emu)**	τ **(s)**	***b***	y_0
8.0	0.0416	35.8(4)	0.444(3)	0.978773(9)
4.0	0.1828	220.8(4)	0.642(1)	0.4514(1)
3.0	0.2146	1092.2(7)	0.5750(3)	0.49824(8)
2.5	0.2211	3104(2)	0.5694(1)	0.57684(9)
2.0	0.2288	10007(7)	0.5140(3)	0.642[b]
1.8[a]	0.2294	12020(10)	0.5312(6)	0.6871(2)

[a] Field decay and field-application were simultaneously fit for this temperature

[b] Field application data was not available at 2K, but the relaxation time is long, causing an unstable fit to (S1). To reduce correlations, y_0 was fixed to a value interpolated from the other temperatures using the Curie law.

Table S6. Relaxation times for DyScS@C_{3v}(8)-C_{82} at 0 T

T (K)	M_0 (m-emu)	τ (s)	*b*	y_0
6.0	0.0173	22(2)	0.64(4)	0.0095(8)
4.0	0.0593	37.8(4)	0.443(2)	0.0187(1)
3.0	0.0831	58.8(4)	0.377(2)	0.0183(2)
2.0	0.1108	97.7(6)	0.283(1)	0.0055(6)
1.8	0.1097	150(1)	0.261(1)	–0.010(1)

Table S7. Relaxation times for DyScS@C_{3v}(8)-C_{82} at 0.3 T

T (K)	M_0 (m-emu)	τ (s)	*b*	y_0
8.0	0.0646	21.6(3)	0.336(3)	0.7748(2)
6.0	0.1153	35.9(5)	0.501(4)	0.5560(2)
5.0	0.1451	85.6(8)	0.516(3)	0.4416(2)
4.0	0.1856	344.0(9)	0.612(2)	0.4393(1)
3.0	0.20898	2001(3)	0.6322(6)	0.5101(2)
2.5	0.2220	5974(6)	0.6174(1)	0.5795(1)
2.0	0.2257	26900(200)	0.5277(4)	0.583(1)

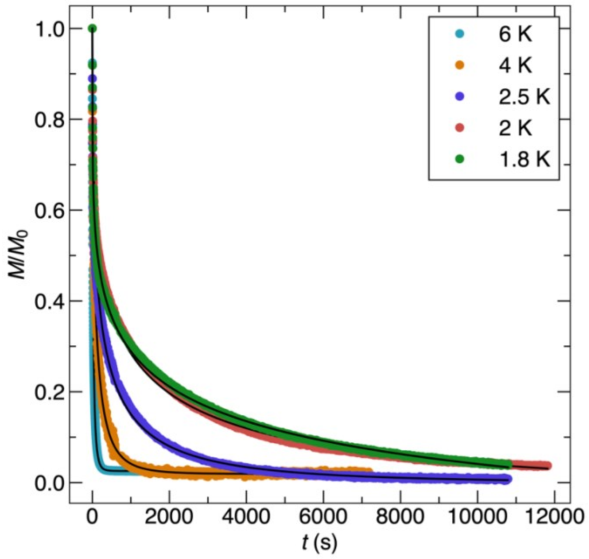

Figure S11. Saturation-decay data with fits to equation S1 (solid lines) for DyScS@C_s(6)-C_{82} at 0 T.

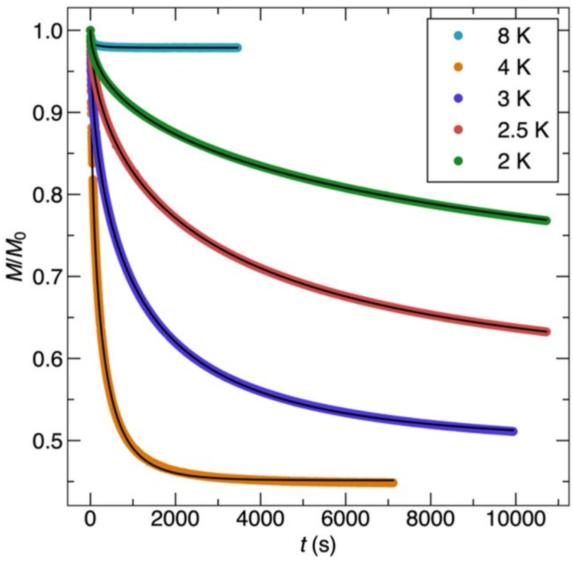

Figure S12. Saturation-decay data with fits to equation S1 (solid lines) for DyScS@C_s(6)-C_{82} at 0.3 T.

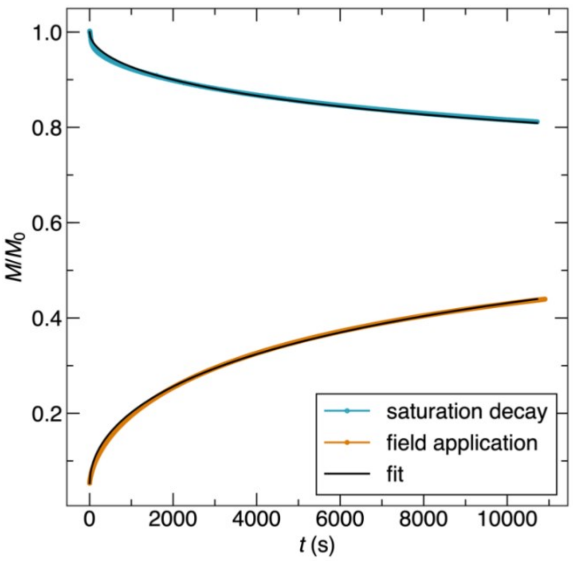

Figure S13. Combined fit of saturation-relaxation and field application data for DyScS@C_s(6)-C_{82} at 0.3 T at 1.8 K.

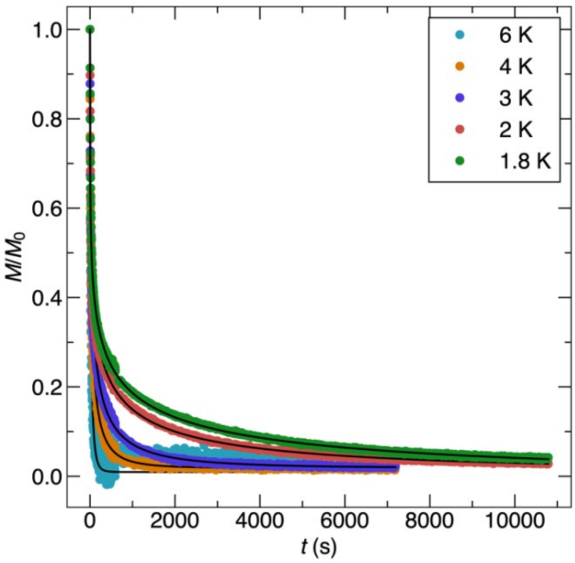

Figure S14. Saturation-decay data with fits to equation S1 (solid lines) for DyScS@C_{3v}(8)-C_{82} at 0 T.

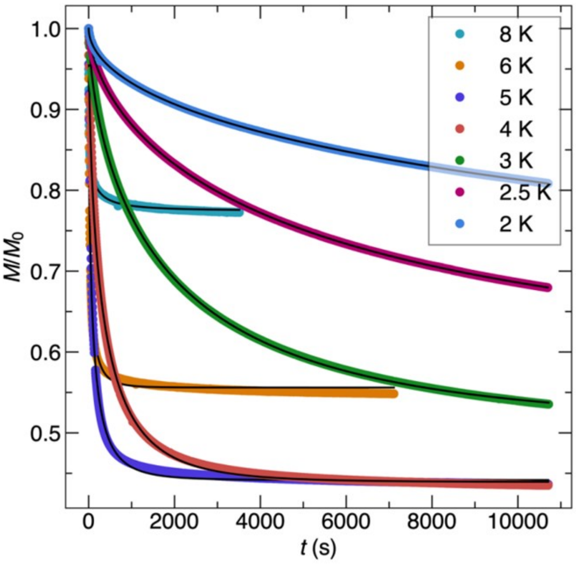

Figure S15. Saturation-decay data with fits to equation S1 (solid lines) for DyScS@C_{3v}(8)-C_{82} at 0 T.

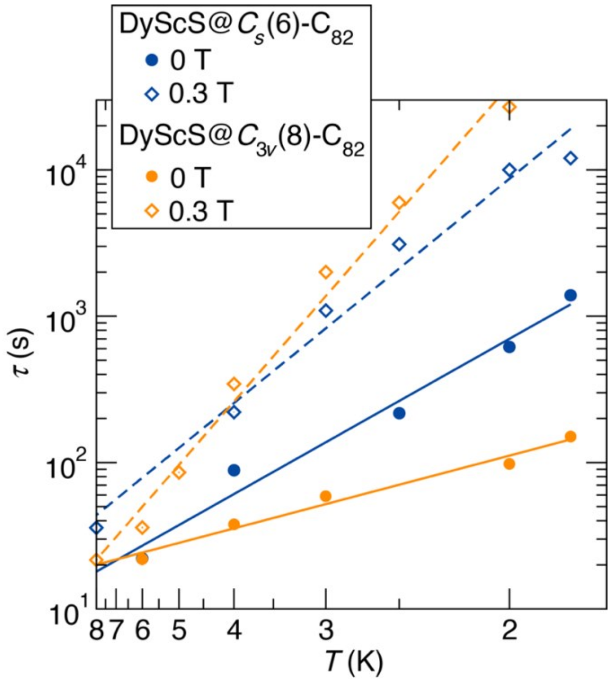

Figure S16. Arrhenius plot of relaxation times for DyScS@C_s(6)-C_{82} and DyScS@C_{3v}(8)-C_{82}, with shown fits to the Orbach relaxation mechanism (main text equation 1). The fit parameters are supplied in Table S8.

Table S8. Results of Orbach fits (main text equation 1) of relaxation time vs. temperature for both isomers of DyScS@C_{82}. Numbers in parenthesis represent the standard deviations from the least-squares fitting.

	H (T)	U_{eff} (cm^{-1})	t_0 (s)
DyScS@C_s(6)-C_{82}	0	6.8(6)	5.3(1.8)
	0.3	9.8(7)	7(3)
DyScS@C_{3v}(8)-C_{82}	0	3.2(3)	11.3(1.7)
	0.3	13.8(7)	1.8(5)

REFERENCES

1. G. M. Sheldrick, *Acta Cryst.*, 2008, **A64**, 112-122.
2. B. Q. Mercado, N. Chen, A. Rodriguez-Fortea, M. A. Mackey, S. Stevenson, L. Echegoyen, J. M. Poblet, M. H. Olmstead and A. L. Balch, *J. Am. Chem. Soc.*, 2011, **133**, 6752-6760.
3. B. Q. Mercado, M. A. Stuart, M. A. Mackey, J. E. Pickens, B. S. Confait, S. Stevenson, M. L. Easterling, R. Valencia, A. Rodriguez-Fortea, J. M. Poblet, M. M. Olmstead and A. L. Balch, *J. Am. Chem. Soc.*, 2010, **132**, 12098-12105.
4. Q. Tang, L. Abella, Y. Hao, X. Li, Y. Wan, A. Rodríguez-Fortea, J. M. Poblet, L. Feng and N. Chen, *Inorg. Chem.*, 2016, **55**, 1926-1933.

5. W. Yang, G. Velkos, F. Liu, S. M. Sudarkova, Y. Wang, J. Zhuang, H. Zhang, X. Li, X. Zhang, B. Büchner, S. M. Avdoshenko, A. A. Popov and N. Chen, *Adv. Sci.*, 2019, **6**, 1970119.
6. N. Chen, M. N. Chaur, C. Moore, J. R. Pinzon, R. Valencia, A. Rodriguez-Fortea, J. M. Poblet and L. Echegoyen, *Chem. Commun.*, 2010 **46**, 4818-4820.
7. C.-H. Chen, D. S. Krylov, Stanislav M. Avdoshenko, F. Liu, L. Spree, R. Yadav, A. Alvertis, L. Hozoi, K. Nenkov, A. Kostanyan, T. Greber, A. U. B. Wolter and A. A. Popov, *Chem. Sci.*, 2017, **8**, 6451-6465.
8. G. Velkos, W. Yang, Y.-R. Yao, S. M. Sudarkova, X. Liu, B. Büchner, S. M. Avdoshenko, N. Chen and A. A. Popov, *Chem. Sci.*, 2020, **11**, 4766-4772.
9. C. Schlesier, L. Spree, A. Kostanyan, R. Westerström, A. Brandenburg, A. U. B. Wolter, S. Yang, T. Greber and A. A. Popov, *Chem. Commun.*, 2018, **54**, 9730-9733.
10. R. Westerstrom, J. Dreiser, C. Piamonteze, M. Muntwiler, S. Weyeneth, H. Brune, S. Rusponi, F. Nolting, A. Popov, S. F. Yang, L. Dunsch and T. Greber, *J. Am. Chem. Soc.*, 2012, **134**, 9840-9843.
11. D. S. Krylov, F. Liu, A. Brandenburg, L. Spree, V. Bon, S. Kaskel, A. U. B. Wolter, B. Büchner, S. M. Avdoshenko and A. A. Popov, *Phys. Chem. Chem. Phys.*, 2018, **20**, 11656-11672.
12. D. S. Krylov, F. Liu, S. M. Avdoshenko, L. Spree, B. Weise, A. Waske, A. U. B. Wolter, B. Büchner and A. A. Popov, *Chem. Commun.*, 2017, **53**, 7901-7904.
13. R. Westerström, J. Dreiser, C. Piamonteze, M. Muntwiler, S. Weyeneth, K. Krämer, S.-X. Liu, S. Decurtins, A. Popov, S. Yang, L. Dunsch and T. Greber, *Phys. Rev. B*, 2014, **89**, 060406.
14. F. Liu, D. S. Krylov, L. Spree, S. M. Avdoshenko, N. A. Samoylova, M. Rosenkranz, A. Kostanyan, T. Greber, A. U. B. Wolter, B. Büchner and A. A. Popov, *Nat. Commun.*, 2017, **8**, 16098.
